# Ice thickness data in the northern sea route (NSR) for the period 2006–2016

**DOI:** 10.1016/j.dib.2019.103925

**Published:** 2019-04-16

**Authors:** Ali Cheaitou, Olivier Faury, Pierre Cariou, Sadeque Hamdan, Gregory Fabbri

**Affiliations:** a Sustainable Engineering Asset Management (SEAM) Research Group and Industrial Engineering and Engineering Management Department, College of Engineering, University of Sharjah, PO. Box 27272, Sharjah, United Arab Emirates; b EM Normandie, Métis Lab, 30 rue Richelieu, 76087 Le Havre Cedex, France; c The Centre of Excellence in Supply Chain (CESIT), Kedge Business School, 680 Cours de la Libération, 33400 Talence, France; d Laboratoire Genie Industriel, CentraleSupélec, Université Paris-Saclay, 91192 Gif-sur-Yvette, France; e Zürich University of Applied Sciences, School of Engineering, Lagerstrasse 41, 8004 Zürich, Switzerland

**Keywords:** Northern sea route (NSR), Copernicus database, Ice thickness variability, Weather conditions, Shipping, Maritime logistics, Ice melting forecasting

## Abstract

This data article includes the datasets of the mean and the standard deviation of ice thickness in a set of sailing zones for a sailing route that goes through the Northern Sea Route (NSR) between Murmansk and Pusan. The route under consideration is between the longitudes 33° 45′ 0″ and 129° 3′ 60″ and the latitudes 69° 24′ 27″ and 35° 6′ 0″ that correspond to the ports of Murmansk (Russia) in the west and Pusan (China) in the east respectively. Within this area, the part that is between the longitude 57° 0′ 0″ and −168° 58′ 0″ and the latitude 70° 27′ 18″ and 69° 6′ 0″ correspond to the NSR. This route has been divided into 49 subzones, and each subzone into squares of 12.5km of side following the data structure of the database Copernicus [1].

The detailed coordinates of the subzones (longitude and latitude) are provided in the article. The daily ice thickness for the period between January 1, 2006 and December 31, 2016 has been obtained for each of the 12.5km sided squares. This data article provides the normality test outcomes and the corresponding p-value of the ice thickness data for each subzone on each calendar day. Moreover, the mean and the standard deviation of the ice thickness in each subzone are also provided. The data provided in this data article can be very helpful for researchers for different applications related to the weather conditions in the NSR zone or to shipping related issues. For instance, the data provided in this paper can be used to investigate the change in ice thickness in the NSR over the period 2006–2016 and to estimate future changes. Another potential application is the estimation of the need for icebreaker assistance as well as the possible ranges for the vessel sailing speed based on the vessel type and for any navigation day in any of the NSR zones. In addition, this data can be used to estimate the risk of blockage for any vessel type because of ice conditions in the NSR zones. It can be helpful to estimate the economic viability of shipping through the NSR since the icebreaker assistance, the speed and the risk of blockage have an effect on the profitability of the shipping lines that may use the NSR.

**Table:** Specifications table

Subject area	*Engineering Management, Economics.*
More specific subject area	*Maritime economics, logistics.*
Type of data	*Tables, MS Excel file (.xlsx), Matlab codes (.m)*
How data was acquired	*Generated using E.U. Copernicus Marine Service Information*[1]*, data set "ARCTIC_REANALYSIS_PHYS_002_003″, available on*http://marine.copernicus.eu/services-portfolio/access-to-products/*.* *A manual that explains how the dataset can be downloaded is available on:*http://cmems-resources.cls.fr/documents/PUM/CMEMS-ARC-PUM-002-ALL.pdf *The license is granted free of charge (worldwide, non exclusive, royalty free, perpetual licence):*http://marine.copernicus.eu/services-portfolio/service-commitments-and-licence/ *Python language has been used to download the files in .nc format (NetCDF). NetCDf4Excel and Matlab have been used to transform the downloaded .nc files to .xlsx files*
Data format	*Analyzed*
Experimental factors	*We looked at the ice thickness for 49 cells on a daily basis over 11 years and took the mean of it.*
Experimental features	*The ARCTIC_REANALYSIS_PHYS_002_003 is a set of data coming from observed data and a numerical model to fill the gap. To do they used the TOPAZ4 system as numerical model.*
Data source location	*Copernicus database, longitudes 33° 45′ 0″ to 129° 3′ 60″ and the latitudes 69° 24′ 27″ to 35° 6′ 0″ that correspond to the zone between the ports of Murmansk (Russia) in the west and Pusan (China) in the east respectively*
Data accessibility	*Data are within this article*
Related research article	Von Schuckmann, K.; Le Traon, P.Y.; Alvarez-Fanjul, E.; Axell, L.; Balmasedam, M.; Breivik, L. A.; Brewin, R. J. W.; Bricaud, C.; Drevillon, M.; Drillet, Y.; Dubois, C.; Embury, O.; Etienne, H.; García Sotillo, M.; Garric, G.; Gasparin, F.; Gutknecht, E.; Guinehut, S.; Hernandez, F.; Juza, M.; Karlson, B.; Korres, G.; Legeais, J.F.; Levier, B.; Lien, V.S.; Morrow, R.; Notarstefano, G.; Parent, L.; Pascual, A.; Pérez-Gómez, B.; Perruche, C.; Pinardi, N.; Pisano, A.; Poulain, P.M.; Pujol, I.M.; Raj, R.P.; Raudsepp, U.; Roquet, H.; Samuelsen, A.; Sathyendranath, S.; She, J.; Simoncelli, S.; Solidoro, C.; Tinker, J.; Tintoré, J.; Viktorsson, L.; Ablain, M.; Almroth-Rosell, E.; Bonaduce, A.; Clementi, E.; Cossarini, G; Dagneaux, Q.; Desportes, C.; Dye, S.; Fratianni, C.; Good, S.; Greiner, E.; Gourrion, J.; Hamon, M.; Holt, J.; Hyder, P.; Kennedy, J.; Manzano-Muñoz, F.; Melet, A.; Meyssignac, B.; Mulet, S.; Buongiorno B.; Nardelli, O'Dea, E.; Olason, E.; Paulmier, A.; Pérez-González, I.; Reid, R.; Racault, M.F.; Raitsos, D.E.; Ramos, A.; Sykes, P.; Szekely, T.; Verbrugge, N. (2017). The Copernicus Marine Environment Monitoring Service Ocean State Report. Journal of Operational Oceanography, Vol. 9 (2), pp. s235-s320. https://doi.org/10.1080/1755876X.2016.1273446.

**Table:** 

**Value of the data** • Ice thickness depends on the weather conditions, which are random by nature, and on the climatic conditions, which are varying over the years. This leads ice-thickness data to have the random variability, and suggests the datasets provided in this data paper to go through a normality test. The p-values of the normality test for ice thickness of the zone between Murmansk (Russia) and Pusan (China) through the NSR are provided in this data paper. The normality test data are provided for 49 subzones and for each calendar day based on the ice thickness data between 2006 and 2016. These data can be used by researchers to check whether ice-thickness is normally distributed or not in a given subzone for a given calendar day and for various application. Considering ice thickness randomness may help researchers in the calculation of the probability of a vessel to be blocked in the NSR because of ice thickness. • The datasets also include the mean and the standard deviation of the ice thickness for the 49 subzones and for any calendar day. Researchers may use these datasets for many applications related to the NSR. For instance, they can be used for weather related investigations, where the data provided in this paper can be compared with future similar datasets to investigate the effect of global warming on ice thickness. • Moreover, the datasets of this data paper can also be used for further analysis on the NSR viability as a shipping route, such as in optimization models. An example of these models can have as objective to find the optimal route to be followed by vessels on the NSR and to minimize the shipping cost or the risk of blockage. • The MATLAB files provided in this data paper can be used by researchers to download and analyze ice thickness data from the database Copernicus [1].

## 1 Data

The datasets of this article provide three categories of data (datasets). The first dataset contains the coordinates of the 49 subzones of the considered route. The second dataset includes two MS Excel files that provide information about the normality test of the daily ice-thickness of all calendar days and the corresponding p-values for 49 subzones based on data from 2006 to 2016. The third dataset contains two other MS files that provide information about the mean and standard deviation of ice-thickness for all calendar days and for the same 49 subzones.

## 2 Experimental design, materials and methods

### 2.1 Data of the route coordinates

The selected sailing route is between the port of Murmansk in Russia in the west and the port of Pusan in China in the east. The route includes the NSR, that is divided into 7 administrative zones by the Russian Administration. Moreover, to obtain more accurate data about ice thickness, the route has been divided into 49 subzones. The coordinates of these subzones, as well as the travelled distance within each subzone, are provided in Table 1. Two figures showing the route on a map are available in the article*.*

**Table 1:** Start and end points and travelled distances in all the subzones of the route.

Subzone number	Longitude	Latitude	Name of the zone	Travelled distance up to the end of the zone [nm]
	33° 45′ 0″ E	69° 24′ 27″ N	**Murmansk port**	6
1	34° 0′ 0″ E	69° 26′ 12″ N	**Murmansk sea**	108
2	39° 0′ 0″ E	69° 55′ 51″ N	Subzone 1.1	125
3	45° 0′ 0″ E	70° 19′ 2″ N	Subzone 1.2	101
4	50° 0′ 0″ E	70° 28′ 49″ N	Subzone 1.3	100
5	55° 0′ 0″ E	70° 30′ 16″ N	Subzone 1.4	59
6	57° 0′ 0″ E	70° 27′ 18″ N	**Kara gate**	65
7	60° 0′ 0″ E	72° 20′ 30″ N	Subzone 2.1	136
8	65° 0′ 0″ E	74° 1′ 20″ N	Subzone 2.2	115
9	70° 0′ 0″ E	75° 27′ 10″ N	Subzone 2.3	100
10	75° 0′ 0″ E	75° 56′ 1″ N	Subzone 2.4	71
11	79° 0′ 0″ E	76° 42′ 28″ N	**Start of Zone 2 of the NSR**	17
12	80° 0′ 0″ E	77° 16′ 29″ N	Subzone 3.1	80
13	85° 0′ 0″ E	77° 36′ 56″ N	Subzone 3.2	74
14	90° 0′ 0″ E	77° 53′ 12″ N	Subzone 3.3	69
15	95° 0′ 0″ E	77° 33′ 22″ N	Subzone 3.4	66
16	100° 0′ 0″ E	77° 48′ 43″ N	Subzone 3.5	64
17	105° 0′ 0″ E	77° 58′ 15″ N	**Vilkitsky strait**	65
18	110° 0′ 0″ E	77° 42′ 35″ N	Subzone 4.1	68
19	115° 0′ 0″ E	77° 20′ 31″ N	Subzone 4.2	73
20	120° 0′ 0″ E	76° 51′ 8″ N	Subzone 4.3	79
21	125° 0′ 0″ E	76° 13′ 7″ N	**Start of Zone 4 of the NSR**	88
22	130° 0′ 0″ E	75° 24′ 37″ N	Subzone 5.1	99
23	135° 0′ 0″ E	74° 22′ 60″ N	Subzone 5.2	115
24	140° 0′ 0″ E	73° 4′ 26″ N	**Sannikov Strait**	87
25	145° 0′ 0″ E	73° 9′ 58″ N	Subzone 6.1	87
26	150° 0′ 0″ E	73° 8′ 14″ N	Subzone 6.2	88
27	155° 0′ 0″ E	72° 59′ 9″ N	Subzone 6.3	90
28	160° 0′ 0″ E	72° 42′ 24″ N	**Start of Zone 6 of the NSR**	94
29	165° 0′ 0″ E	72° 17′ 23″ N	Subzone 7.1	99
30	170° 0′ 0″ E	71° 43′ 10″ N	Subzone 7.2	106
31	175° 0′ 0″ E	70° 58′ 25″ N	Subzone 7.3	115
32	180° 0′ 0″ E	70° 1′ 12″ N	**Wrangel Island**	104
33	175° 0′ 0″ W	69° 49′ 45″ N	Subzone 8.1	106
34	170° 0′ 0″ W	69° 29′ 24″ N	Subzone 8.2	67
35	168° 58′ 0″ W	69° 6′ 0″ N	**End of the NSR**	303
36	169° 14′60″ W	64° 5′ 60″ N	**Bering Strait**	33
37	170° 0′ 0″ W	64° 4′ 13″ N	Subzone 9.1	139
38	175° 0′ 0″ W	63° 22′ 51″ N	Subzone 9.2	156
39	180° 0′ 0″ E	62° 8′ 8″ N	Subzone 9.3	171
40	174° 59′ 60″ E	60° 34′ 52″ N	Subzone 9.4	191
41	170° 0′ 0″ E	58° 39′ 11″ N	Subzone 9.5	216
42	164° 59′ 60″ E	56° 15′ 59″ N	Subzone 9.6	248
43	160° 0′ 0″ E	53° 18′ 27″ N	Subzone 9.7	289
44	155° 0′ 0″ E	49° 37′ 45″ N	Subzone 9.8	198
45	152° 0′ 0″ E	47° 0′ 0″ N	**Pacific**	83
46	150° 0′ 0″ E	46° 48′ 20″ N	Subzone 10.1	210
47	145° 0′ 0″ E	46° 9′ 28″ N	Subzone 10.2	131
48	141° 56′ 36″ E	45° 38′ 41″ N	**Japan strait**	862
49	129° 3′ 60″ E	35° 6′ 0″ N	**Pusan port**	6

### 2.2 Downloading the data of the ice thickness

Each subzone defined in Table 1 has been defined using its coordinates in the database Copernicus [1]. Matlab has then been used to call Python and download the data from the Copernicus server automatically for all coordinates and for all the dates between the 1st of January 2006 and the 31st of December 2016 which corresponds to 11 years of data. Since the structure of the database Copernicus [1] divides the defined zone into squares of 12.5km of side, then the downloaded data for a given zone and a given date will contain as many values as the number of squares in the zone for a given parameter. The exact dataset that was used from Copernicus is ARCTIC_REANALYSIS_PHYS_002_003 and its subset dataset-ran-arc-day-myoceanv2-be which requires defining the coordinates of the zone for which the ice thickness data is required, and the required range of dates. It then allows the user to define the parameters that are required to be downloaded, including the water temperature, salinity, ice thickness, depth, etc. We selected only the data for the ice thickness. A total of 196,882 files have been downloaded corresponding to 11 years, 365 days (or 366 days) in each year and 49 zones. We used four computers in parallel to speed up the download process of the data, where each computer was responsible for downloading the data for part of the zones and for the 11 years. In addition, the cores of the processor of each computer were used in parallel to download different files at the same time using the "*parfor*" loop of Matlab. The download request through the website server took between 20 seconds and 2 minutes per file on every computer, with the majority of the cases being in the low range (close to 20 seconds per file) which corresponded to a total download time of around 11 days.

The downloaded files are in the format .nc (NetCDF). They result from the TOPAZ system based on an advanced sequential data assimilation method and the Hybrid Coordinate Ocean Model (HYCOM version 2.2). The dataset includes 26-years Arctic reanalysis product in the period 1991–2017 included. The variables delivered are all physical variables, including 3D currents, temperatures and salinities, 2D parameters for sea ice, mixed layer depth and sea surface heights. Sea surface temperature and sea surface heights are corrected for bias, with an online bias correction algorithm. Table 2 provides the metadata of the dataset.

**Table 2:** Meta data descriptors of the data available in the dataset "ARCTIC_REANALYSIS_PHYS_002_003".

Geographical coverage	−180W, 180E, 62N, 90N
Spatial resolution	12.5 km × 12.5 km
Vertical coverage (m)	From −3000 to 0 (12 levels)
Coordinate reference system	NSIDC sea ice polar stereographic north (EPSG 3411)
Feature type	Grid
Temporal coverage	From 1991 to 01-01 to 2017-12-31
Areas	Arctic-ocean
Observation/models	Numerical model
Temporal resolution	Monthly-mean Daily-mean
UPDATE FREQUENCY	Annually (undefined)
Variables	sea_water_potential_temperature (T)
	sea_water_temperature (T)
	sea_water_potential_temperature_at_sea_floor (bottomT)
	sea_water_salinity (S)
	sea_surface_height_above_sea_level (SSH)
	sea_water_x_velocity (3DUV)
	sea_water_y_velocity (3DUV)
	ocean_mixed_layer_thickness_defined_by_sigma_theta (MLD)
	sea_ice_area_fraction (SIC)
	sea_ice_thickness (SIT)
	sea_ice_x_velocity (SIUV)
	sea_ice_y_velocity (SIUV)
	surface_snow_thickness (SNOW)
	sea_water_potential_temperature (T)

It is worth noting that ARCTIC_REANALYSIS_PHYS_002_003 is a set of data coming from observed data and a numerical model to fill the gap. It is a merged product of weekly sea ice thickness (SIT) measurements in Arctic from the satellite CryoSat-2 altimeter and the satellite Soil Moisture and Ocean Salinity (SMOS) radiometer (referred to as CS2SMOS). This product is gridded with a resolution of approximately 25 km [2]. The database includes an estimate of the observation error but it only accounts for the errors related to the merging and interpolation [3]. A method has been used to evaluate the observation error suitable in the TOPAZ4 system for assimilating CS2SMOS data in a short sensitivity experiment, to which a term to the C2SMOS raw error estimate has been added. The amplitude of SIT has increased to ε = min (0.5, 0.1+0.15*d), where d represents the merged SIT measurement. The aforementioned maximal observation error is limited by a threshold value of 0.5 m in the years of 2014–2015 only. Afterwards, the additional observation error term has been tuned as ε = min (0.25, 0.1+0.075*d) for the winter 2016–2017.

The total size of the downloaded files is 24.79 GB. The detailed file sizes per zone are shown in Table 3.

**Table 3:** Size of the NetCDF data files for the 49 zones.

Subzone number	Total size of the NetCDF size [bytes]	Subzone number	Total size of the NetCDF size [bytes]
1	9,836,048	26	12,503,848
2	16,634,104	27	15,027,000
3	16,232,328	28	15,910,904
4	12,053,864	29	18,176,920
5	11,909,224	30	21,712,544
6	11,041,384	31	23,673,208
7	14,609,152	32	28,864,144
8	32,287,272	33	7,895,490,744
9	26,678,488	34	22,725,016
10	22,725,016	35	15,139,496
11	17,116,232	36	94,578,504
12	10,414,608	37	10,591,392
13	18,562,624	38	34,633,640
14	16,457,320	39	15,333,985,512
15	15,235,920	40	57,470,544
16	14,609,152	41	68,527,400
17	14,046,664	42	85,080,536
18	13,419,896	43	108,769,200
19	14,480,584	44	138,291,640
20	14,978,784	45	68,270,264
21	15,669,840	46	15,428,776
22	16,987,664	47	42,910,216
23	18,434,056	48	23,560,712
24	20,925,064	49	226,264,352
25	12,503,848		

Matlab has built-in function to read the NetCDF files. However, for the data files that have been included in this data paper, this functionality was able to read part of the data correctly but not the ice thickness data. Therefore, to avoid any risk of having incorrect data, NetCDF4Excel macro was utilized after modifying it to be connected with Matlab. We therefore, converted the NetCDF files into MS Excel files. To do so, we modified the Excel Macro (NetCDf4Excel) so that it opens the NetCDF files without requiring the user to browse for the file. Instead, this macro was modified to require the file name only while calling it, i.e. without browsing. Matlab has then been used to call the Macro in an automatic way and to feed it with the file name so that the file can be read in Matlab and then saved as an MS Excel file. The file names have been generated in an automatic way in Matlab in order to fit with the file names that are given by the database Copernicus. The complete process has then been done automatically. Indeed, the file names are generated automatically based on the zone number and the date. Each MS Excel file is stored in the folder that belongs to its zone. An iterative approach (using loops) has been used to automate the process. In addition, the NetCDF4Excel macro asks the user to browse and select the .nc file and then the user needs to save the converted file manually to MS Excel (.xlsx format). To automate things, this NetCDF4Excel macro has been modified by replacing the "browse" command with a variable that contains the file name and path. In addition, a code has been added to automatically save the converted file to .xlxs file. Excel is called using the command actxserver ('Excel.Application'). This command creates a Microsoft Component Object Model (COM) that can be used to control MS Excel through Matlab.

### 2.3 Normality test and p-value of ice thickness

First, some of the ice thickness data that were downloaded had small very small negative values (all of them close to zero). The downloaded data have then been processed in order to eliminate these negative values by setting all the negative values to be equal to zero.

Given the variability of the ice thickness data, normality test has been conducted. Lilliefors test was performed using the Matlab function lillietest () that returns the selected hypothesis (0 for H_0_ if the data are normally distributed or 1 for H_1_ if the data are not normally distributed) and the corresponding p-value. To perform the test for a given subzone and a given date, the ice thickness data of each square of 12.5 km of side in the subzone and for the considered date have been first obtained and averaged. For example, for 1 January, the average ice thickness data of 1/1/2006 (based on all the squares of the considered subzone), the average of 1/1/2007, 1/1/2008 … etc. have been calculated. Therefore, 11 means (from 11 years) have been obtained for each subzone. The Lilliefors test has then been used on these 11 data points for each day and each subzone. The corresponding outcomes are two 365 by 49 cells MS Excel files that are provided in the supplementary materials of this data paper and are described in Table 4.

**Table 4:** Ice thickness normality analysis data files description.

File name	File type	Description
Normality_Test.xlsx	MS Excel	A single sheet MS Excel file with 365 rows corresponding to the calendar days and 49 columns corresponding to the subzones. The cells contain one of the three following values: - 0: which means that the null hypothesis is not rejected and therefore the data are normally distributed - 1: which means that the null hypothesis is rejected and therefore the data are not normally distributed. - NaN: which means that the test is not applicable.
p_value.xlsx	MS Excel	A single sheet MS Excel file with 365 rows corresponding to the calendar days and 49 columns corresponding to the subzones. The cells contain the p-value of the normality tests for each corresponding subzone and calendar day.

It is worth noting that out of 17,885 tests, for 5,253 tests the null hypothesis was rejected which means that for the majority of the tests the null hypothesis was accepted and therefore the ice thickness data can be considered as normally distributed for each subzone and each calendar day.

### 2.4 Ice thickness mean and standard deviation

Based on the normality tests, the mean and standard deviations of the ice thickness for the 11 values described in section 2.2 for each subzone and for each calendar day have been calculated. The obtained data are provided in the supplementary materials of this data paper and are described in Table 5.

**Table 5:** Ice thickness mean and standard deviation data files description.

File name	File type	Description
Mean.xlsx	MS Excel	A single sheet MS Excel file with 365 rows corresponding to the calendar days and 49 columns corresponding to the subzones. The cells contain the mean of ice thickness of the considered subzone and calendar day over the period 2006–2016.
Standard_Deviation.xlsx	MS Excel	A single sheet MS Excel file with 365 rows corresponding to the calendar days and 49 columns corresponding to the subzones. The cells contain the standard deviation of ice thickness of the considered subzone and calendar day over the period 2006–2016.

### 2.5 Matlab scripts

To download the data using python, motu client package is required. Table 6 includes the Matlab code file names as well as their descriptions. These files are the ones that are used to download the data.

**Table 6:** Matlab files used for the download of the ice thickness data.

Script file	Description
Download_From_Server_P1	This script takes the following input: 1 The path of python motu-client package 2 The Copernicus account details (username and password) 3 The excel file contains the zones coordiantes 4 The database specific details (Mercator_motu_web, service_id, and product_id) The script prepares the python commands to download the data files (NetCDF) for all zones and all dates and provides two output files (.mat): 1 Download commands as a string 2 The full path where the data file will be saved for each date and each zone
Download_From_Server_P2	This script loads the data file produced by Download_From_Server_P1 and evaluates the python command (which was saved as string) and starts the downloading process. This script uses “parfor” loop which utilizes the parallel computing to download more than one file at the same time. This script was run on different computers to speed up the process where each computer was responsible for downloading part of the zones.
Find_Missing_Files	This script loads the data files produced by Download_From_Server_P1 and compares the downloaded files (Download_From_Server_P2) with the list of files to be downloaded (Download_From_Server_P1). If a file is identified as missing then the script downloads it. A file might be missing due to some server issues during the downloading process.
Convert_Nc_to_xlsx	This file requires the full path (generated by Download_From_Server_P1) and stored in the (.mat file). In addition, it requires the path where the modified NetCDF4Excel exists. It calls and opens the modified macro and converts the NetCDF files to .xlsx files and stores them using the same file name of the NetCDF files.
